# Consequences of Asexuality in Natural Populations: Insights from Stick Insects

**DOI:** 10.1093/molbev/msy058

**Published:** 2018-04-05

**Authors:** Jens Bast, Darren J Parker, Zoé Dumas, Kirsten M Jalvingh, Patrick Tran Van, Kamil S Jaron, Emeric Figuet, Alexander Brandt, Nicolas Galtier, Tanja Schwander

**Affiliations:** 1 Department of Ecology and Evolution, University of Lausanne, Lausanne, Switzerland; 2 Swiss Institute of Bioinformatics, Lausanne, Switzerland; 3 Institute of Evolutionary Sciences, University of Montpellier, CNRS, IRD, EPHE, Montpellier, France; 4 Johann-Friedrich-Blumenbach Institute of Zoology and Anthropology, University of Goettingen, Goettingen, Germany

**Keywords:** evolution of sex, parthenogenesis, polymorphism, purifying selection, biased gene conversion, *Timema*

## Abstract

Recombination is a fundamental process with significant impacts on genome evolution. Predicted consequences of the loss of recombination include a reduced effectiveness of selection, changes in the amount of neutral polymorphisms segregating in populations, and an arrest of GC-biased gene conversion. Although these consequences are empirically well documented for nonrecombining genome portions, it remains largely unknown if they extend to the whole genome scale in asexual organisms. We identify the consequences of asexuality using de novo transcriptomes of five independently derived, obligately asexual lineages of stick insects, and their sexual sister-species. We find strong evidence for higher rates of deleterious mutation accumulation, lower levels of segregating polymorphisms and arrested GC-biased gene conversion in asexuals as compared with sexuals. Taken together, our study conclusively shows that predicted consequences of genome evolution under asexuality can indeed be found in natural populations.

## Introduction

The absence of recombination is a common feature in eukaryotic genomes and it can span over a few hundred bases, entire chromosomes and, as in the case of asexual organisms, over complete genomes. The reasons for an absence of recombination can vary. For example, centromeric regions do not recombine because of their mechanistic role as the attachment points for kinetochores during meiosis (Talbert and Henikoff 2010). Recombination can be reduced in genome portions with nearby transposable elements as a consequence of heterochromatin formation through local epigenetic silencing (Castel and Martienssen 2013), or because of extensive structural differences between homologous chromosomes, a trait notably characterizing many supergenes and sex chromosomes (Bachtrog 2013; Schwander et al. 2014). Finally, meiotic recombination is completely lacking in asexual organisms because of clonal reproduction.

Theory predicts two major consequences for selection on genome portions and complete genomes that lack recombination. First, selection is less effective, because physical linkage among loci hinders selection’s ability to act upon loci independently (Muller 1964; Hill and Robertson 1966; Felsenstein 1974; Keightley and Otto 2006). This should translate into decreased rates of adaptation and increased accumulation of mildly deleterious mutations, including point mutations and repetitive elements. A related second consequence is that population levels of polymorphisms change (Hill and Robertson 1966; Charlesworth et al. 1993). Whether the lack of recombination increases or decreases polymorphism depends on the relative importance of multiple counteracting factors, and especially the level of heterozygosity in the nonrecombining region. A classical prediction is that heterozygosity (and thus polymorphism) increases over time in the absence of recombination, as allelic sequences diverge independently of each other (Birky 1996). Polymorphism levels can therefore be higher in nonrecombining than recombining genomes and genome portions (Balloux et al. 2003). In contrast, mitotic gene conversion and selection at linked sites (background selection) lead to a loss of heterozygosity and diversity in the absence of recombination, which can translate to lower polymorphism in recombining than nonrecombining genome portions (Hill and Robertson 1966; Charlesworth et al. 1993).

In addition to selection-driven consequences of linkage, mechanistic effects that are independent of selection or drift can differ between recombining and nonrecombining genome portions. For example, recombination per se is thought to be mutagenic, which can affect polymorphism levels and the rate of divergence (Webster and Hurst 2012). Finally, base compositions are expected to differ between recombining and nonrecombining genomes and genome portions because recombination-associated gene conversion will tend to increase GC content. Gene conversion is a molecular mechanism associated with DNA double-strand breaks and mismatch repair during recombination, where one allele is replaced by its homolog (van den Bosch et al. 2002). In many species, gene conversion is nonrandom with respect to the base composition of an allele; the bases A and T are more likely to be converted into G and C than the reverse (Marais 2003; Mugal et al. 2015). In the absence of meiotic recombination, such GC-biased gene conversion (gBGC) should occur at greatly reduced rates, and the base composition of neutral sequences should shift towards the mutational equilibrium (Galtier et al. 2018). There is accumulating empirical evidence for several of the above mentioned consequences in nonrecombining genome portions of sexual organisms. For instance, studies of Y-chromosomes revealed lower levels of neutral polymorphism, increased rates of deleterious mutation accumulation and lower rates of adaptive evolution compared with recombining parts of genomes (Bachtrog and Charlesworth 2002; Skaletsky et al. 2003; Bachtrog 2013). Furthermore, as a result of reduced gBGC, nonrecombining genome portions typically feature low GC content (Spencer et al. 2006; Duret and Galtier 2009; Pessia et al. 2012; Glémin et al. 2015).

In contrast to these nonrecombining, “asexual” genome portions in sexual genomes, the consequences of a genome-wide lack of recombination in natural populations of asexual species remain less clear. In particular, the effects of asexuality on segregating polymorphisms and gBGC remain largely untested. The reduced effectiveness of selection in asexuals was investigated in several studies via analyses of deleterious mutation accumulation, but with mixed results. Most studies based on few individual genes indeed found increased rates of mutation accumulation in asexual as compared with sexual lineages, but others reported no differences (reviewed in Hartfield 2016). Similarly, the three available genome-wide studies all reported different patterns, including one case where selection was more effective in asexual than sexual species (Hollister et al. 2015; Ament-Velásquez et al. 2016; Brandt et al. 2017). Given these conflicting results, additional whole-genome studies are warranted.

**Fig. 1.**
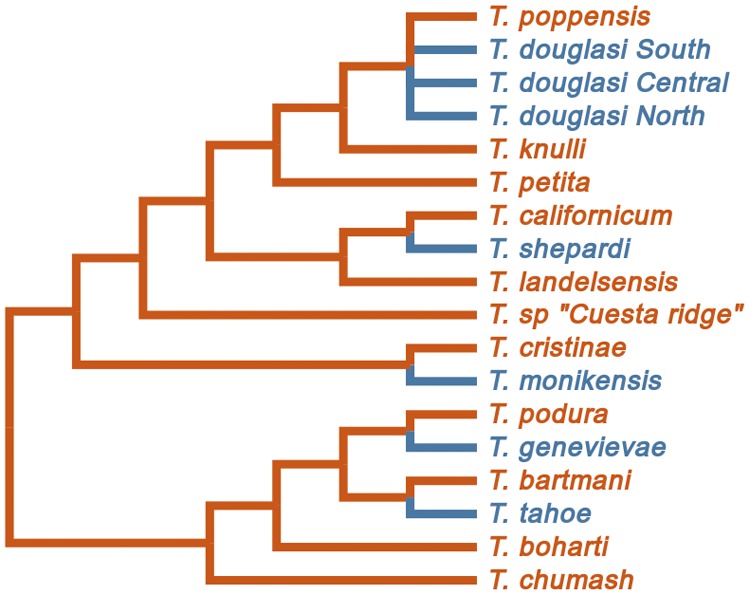
Phylogeny of *Timema* species (sexual species phylogeny redrawn from Riesch et al. 2017; asexual lineages added from Schwander et al. 2011). Sexual species are colored red, asexual species blue. For this study, five sex–asex species pairs were used (for *Timema douglasi* only the southern lineage).

*Timema* stick insects are an ideal group for identifying signatures of asexual genome evolution. Because asexuality is a lineage level-trait, independently derived asexuals are required to disentangle consequences of asexuality from species-specific factors. Seven such independently derived asexuals are known in *Timema*, with an ecologically equivalent sexual sister species at hand for comparison (fig. 1). Sexual and asexual sister species are morphologically alike, live and feed on similar host plants and have a generation time of one year (Law and Crespi 2002). The asexual lineages are obligate apomicts, diploid, not of hybrid origin and differ in age, ranging from 0.2 to 2 My (Schwander and Crespi 2009; Schwander et al. 2011).

Here, we tested whether the lack of recombination in *Timema* asexuals is associated with 1) changes in the strength of gBGC; 2) changes in the amount of genetic variation within populations; and 3) increased rates of deleterious mutation accumulation and more segregating deleterious variants. We generated de novo transcriptomes for ten *Timema* species (see Materials and Methods for details), including transcriptomes of five asexual lineages (*T. tahoe*, *T. monikensis*, *T. douglasi “South,” T. shepardi*, and *T. genevievae*), and their sexual sister-species (*T. bartmani*, *T. cristinae*, *T. poppensis*, *T. californicum*, and *T. podura*).

## Results

### De novo Transcriptome Assembly and Ortholog Prediction

Depending on the species, the quality-filtered assemblies contained between 31,747 and 45,655 transcripts with a N_50_ between 1,301 and 1,836 bp. Open reading frames (ORFs) were identified in 7,329–10,436 of the transcripts, and these ORFs were used to determine orthologs shared between the two species of a pair (between 5,329 and 5,908 per pair) as well as orthologs shared across all ten species (3,010 orthologs; see Materials and Methods for details, supplementary data 1 and supplementary tables 1 and 2, Supplementary Material online).

**Fig. 2.**
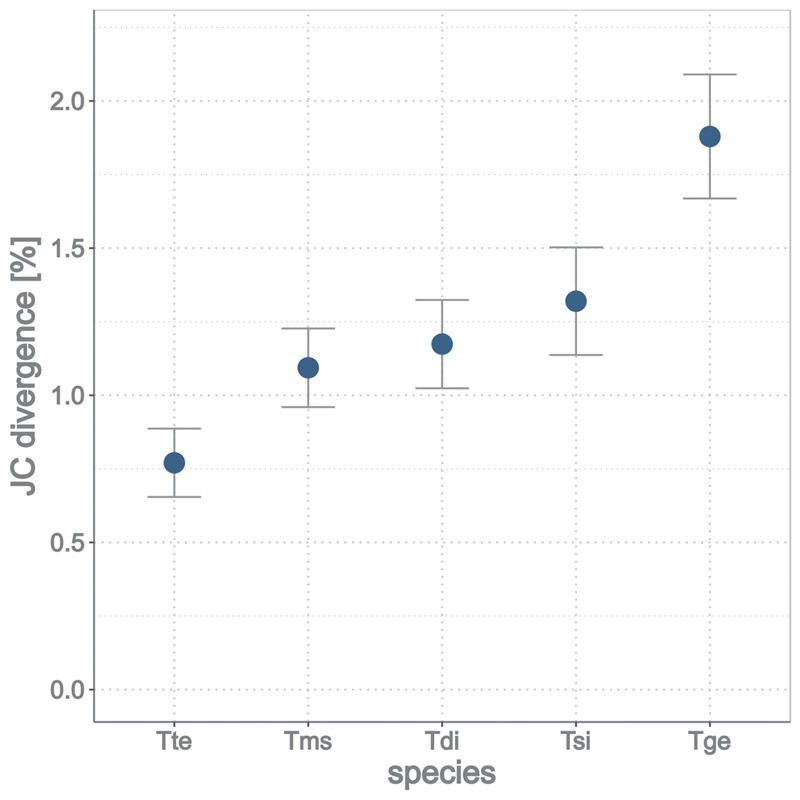
Asexual lineages ranked from youngest to oldest as estimated from Jukes–Cantor corrected divergence between sexual–asexual sister-species (depending on the species pair, using 5,329–5,908 pairwise orthologs). Tte: *Timema tahoe*, Tms: *Timema monikensis*, Tdi: *Timema douglasi*, Tsi: *Timema shepardi*, Tge: *Timema genevievae*. The oldest asexual lineage *Timema genevievae*, was previously estimated to be ∼1.5 My old (Schwander et al. 2011).

The ages of the asexual lineages were previously estimated based on intra and interspecific divergence at a single mtDNA locus (Schwander et al. 2011). We used orthologs within each species-pair to update the ranking of asexuals from youngest to oldest, with interspecific divergence as a proxy for lineage age (fig. 2).

To evaluate the robustness of the 3,010 ten-species orthologs, we screened each transcriptome for variants of these orthologs. Variants are transcripts with the same ORF, but that contain sufficiently many SNPs, deletions or insertions of variable length in any part of the ORF to result in separately assembled transcripts in the de novo assembly (our ortholog detection pipeline retained the longest ortholog if multiple options are available, see Materials and Methods for details). Surprisingly, the proportion of orthologs with variants was significantly higher for the asexual than sexual *Timema* species (mean asex: 0.076, mean sex: 0.013; paired *t*-test, *t* = 7.29, df = 4, *P* < 0.001; supplementary data 2, Supplementary Material online). Given this systematic difference between sexual and asexual species, we evaluated whether ortholog variants represent transcripts from multiple gene copies by mapping genomic reads from single individuals generated in a different study (SRR5248877–SRR5248936) to our 3,010 ten-species orthologs (see Supplementary Material online). If ortholog variants were generated by gene duplications, the coverage for orthologs with variants would be higher than the coverage for orthologs without variants (i.e., an ortholog with two copies in the genome would have twice the coverage relative to an ortholog with single copy). Our analyses revealed that ortholog variants do not stem from gene duplicates, as the coverage for orthologs was the same, independently of whether an ortholog had a variant or not in a given transcriptome (supplementary fig. 1, Supplementary Material online). By consequence, ortholog variants are most likely generated by transcriptional noise and error, or perhaps by different alleles (see discussion for details). Indeed half of the variants (725 out of 1,353) differed in length from each other (up to 7-fold, mean length difference: 13%) and 27% of the variants further featured SNP divergences (mean pairwise distance 1.55%). While the enrichment of orthologs with variants in asexuals is interesting per se, it is important to note that these variants did not affect our subsequent analyses and results. This is because all polymorphism, pN/pS and dN/dS analyses were based on one “best” ortholog variant (i.e., longest) and estimates of segregating polymorphism were conservative, as the presence of variants could potentially inflate polymorphisms in asexuals compared with sexuals but sexuals had more polymorphisms than asexuals (see below).

### Reduced gBGC in Asexuals

**Fig. 3.**
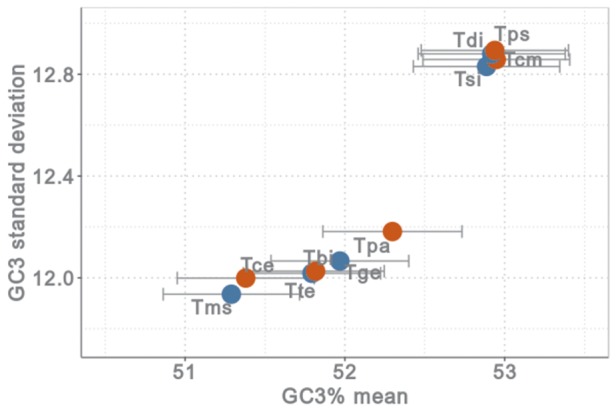
Mean GC3 content (with 95% CI) and GC3 standard deviation of the 3,010 ten-species orthologs of sexual (in red) and asexual (in blue) *Timema* species. Tbi: *T. bartmani*, Tte: *T. tahoe*, Tce: *T. cristinae*, Tms: *T. monikensis*, Tps: *T. poppensis*, Tdi: *T. douglasi*, Tcm: *T. californicum*, Tsi: *T. shepardi*, Tpa: *T. podura*, Tge: *T. genevievae*.

Because meiotic recombination has stopped in asexuals, gBGC should be strongly reduced if the process was at work in *Timema*. We tested for evidence of this mechanistic consequence of asexuality via various approaches. First, we analyzed GC content at third codon positions (GC3 content), which are most likely neutral. In agreement with the hypothesis that gBGC favors the increase of GC content and generates variation among genes experiencing different recombination rates, sexual species within each pair had a higher GC3 content (Wilcoxon signed-rank test, *P* < 0.01 in all pairs) and higher variance among genes than asexual species (fig. 3). We also compared per gene GC3 in each of the sexual–asexual species pairs. We calculated the proportion of genes in which GC3 was higher in the sexual than in the asexual species, and found that in all five pairs this proportion was above 0.5. Interestingly, the proportion was maximal (0.62) in the oldest pair (*T. podura* vs. *T. genevieve*), minimal (0.51) in the youngest pair (*T. bartmani* vs. *T. tahoe*), and intermediate in the other three pairs (0.52, 0.54, 0.55). Then we investigated the substitution processes that shaped base composition in *Timema* using maximum likelihood approaches. We estimated branch-specific equilibrium GC3 using the nonhomogeneous maximum likelihood method developed by Galtier and Gouy 1998. Similar to the results from the previous approach, the asexual species were characterized by a lower equilibrium GC3 than their sexual counterparts (likelihood ratio test, *P* < 2.2e-16 between stationary and nonstationary per-branch model of sequence evolution), meaning that current substitution processes drive GC3 towards lower values in asexuals than in sexuals. We mapped synonymous substitutions along the *Timema* phylogeny and estimated the per-lineage number of weak-to-strong (A/T → G/C), strong-to-weak (G/C → A/T), and GC-conservative (A ↔ T, G ↔ C) substitutions. As expected under gBGC arrest, asexuals always harbored a higher proportion of strong-to-weak substitutions and a lower proportion of weak-to-strong substitutions than to sexuals (supplementary data 3, Supplementary Material online). Finally, we calculated GC-content in UTR regions and correlated it to GC3. We obtained a strong and significantly positive correlation between GC_UTR and GC3 in all ten species (*r* between 0.37 and 0.44, *P *< 0.001 in all cases), demonstrating that GC3 is governed by evolutionary forces independent of the process of translation—most likely gBGC. In combination, the results from these different approaches strongly support the hypothesis of a reduced impact of gBGC in *Timema* asexuals.

### Reduced Levels of Polymorphisms in Asexuals

**Fig. 4.**
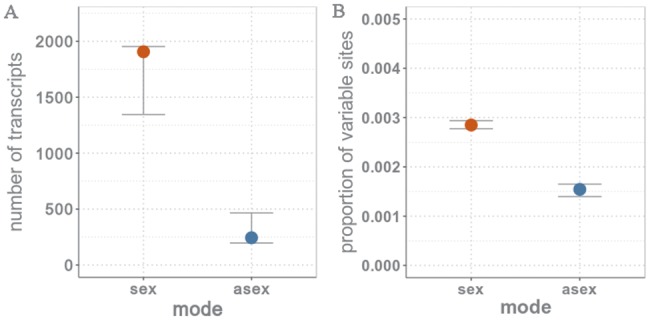
Medians (and 95% CI with 5,000 bootstrap replicates) of (*A*) number of transcripts containing SNPs and (*B*) proportion of variable sites per transcript for sexual (red) and asexual (blue) *Timema* species.

Polymorphism levels at individual loci can be higher or lower in asexual than sexual populations, depending on the amount of heterozygosity in asexuals and the number of different clones (genotype diversity). To quantify the amount of segregating polymorphisms in sexuals and asexuals, we identified SNPs in transcripts by mapping the read data derived from three pooled individuals (all collected from the same population) back to the 3,010 ten-species orthologs that include only one “best” variant per ortholog (see Materials and Methods for details). Since alternative variants of each ortholog were not included, reads corresponding to these variants mapped to the “best” ortholog. Specifically, for 63% of the orthologs, the sum of reads corresponding to the different variants was exactly identical to the number of reads mapping to the best ortholog; for 93%, the read numbers differed by 5% or less (supplementary fig. 2, Supplementary Material online). As expected given the larger number of variants in asexuals than sexuals, mapping quality was somewhat lower in asexuals than sexuals, however not significantly so (paired *t*-test, *t* = 1.3, df = 7.9, *P* = 0.215). We found that a higher proportion of transcripts contained SNPs in sexuals than asexuals (paired *t*-test, *t* = 13.46, df = 4, *P* < 0.001; fig. 4*A*), in spite of the enrichment for ortholog variants in asexual transcriptomes. Similarly, among these transcripts that contained SNPs, the proportion of variable sites per transcript was higher for sexuals than asexuals (paired *t*-test, *t* = 3.850, df = 4, *P* = 0.018; fig. 4*B*).

### Purifying Selection is Less Effective in Asexuals

To test the prediction that selection acts less effectively on asexuals than sexuals, we focused on mutations that are expected to affect protein function and are likely deleterious. This should be the case for nonsynonymous mutations in the 3,010 orthologs shared among the ten species. Indeed, since the *Timema* species diverged ∼30 Ma, the vast majority of these orthologs should be under purifying selection (or they would be too diverged to be identified as orthologs). Thus, we quantified whether more nonsynonymous mutations segregate in asexual than sexual populations and whether these mutations accumulate faster in asexual than sexual species.

**Fig. 5.**
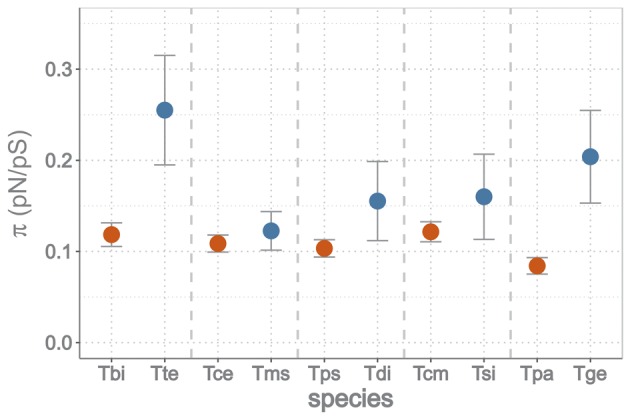
Means (and 95% CI) of nonsynonymous segregating polymorphisms (pN/pS) of sexual (in red) and asexual (in blue) *Timema* species. Tbi: *T. bartmani*, Tte: *T. tahoe*, Tce: *T. cristinae*, Tms: *T. monikensis*, Tps: *T. poppensis*, Tdi: *T. douglasi*, Tcm: *T. californicum*, Tsi: *T. shepardi*, Tpa: *T. podura*, Tge: *T. genevievae*. Species pairs are ranked by age of the asexual lineage (youngest to oldest).

We identified nonsynonymous and synonymous SNPs per transcript by mapping the read data (derived from three pooled individuals) to the 3,010 ten-species orthologs (see Materials and Methods). As expected, the ratio of nonsynonymous to synonymous polymorphisms (pN/pS) was larger in asexual than sexual populations (paired *t*-test, *t* = −3.02, df = 4, *P* = 0.039; fig. 5). This indicates that deleterious variants are rapidly removed by selection in sexual populations but persist longer in asexual populations.

**Fig. 6.**
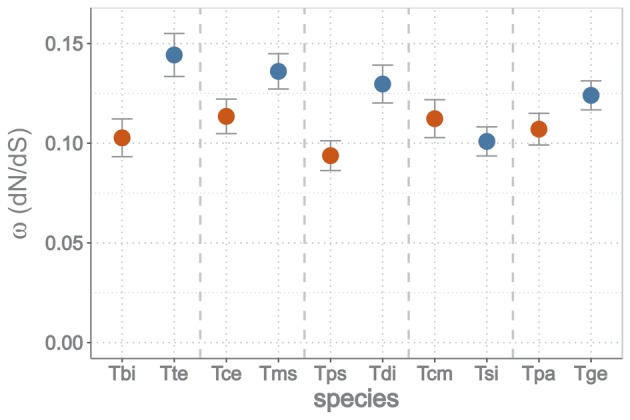
Means (and 95% CI) of *ω* = dN/dS ratios from 2,487 orthologs in sexual (in red) and asexual (in blue) *Timema* species. Tbi: *T. bartmani*, Tte: *T. tahoe*, Tce: *T. cristinae*, Tms: *T. monikensis*, Tps: *T. poppensis*, Tdi: *T. douglasi*, Tcm: *T. californicum*, Tsi: *T. shepardi*, Tpa: *T. podura*, Tge: *T. genevievae*. Species pairs are ranked by age of the asexual lineage (youngest to oldest).

To elucidate whether deleterious variants also become fixed faster in asexual than sexual *Timema* species, we estimated the ratio of nonsynonymous to synonymous divergence (*ω* = dN/dS) along the branches of the *Timema* phylogeny, using the maximum likelihood methods implemented in PAML (Yang 2007; see Materials and Methods for details). Again, for transcripts under purifying selection (i.e., the majority of the ten-species orthologs), most nonsynonymous changes are likely mildly deleterious (Li et al. 1985) and a higher *ω* ratio therefore indicates a higher rate of nonsynonymous mutation accumulation and less effective purifying selection. As the *Timema* system provides evolutionary replicates, we allowed *ω* to vary independently for each gene and branch on the phylogeny (i.e., applying a “free model”), to take these replicates into account. Out of the 3,010 ten-species orthologs, we removed 200 without variation and 323 with *ω* ≥ 1 or without synonymous substitutions, which left 2,487 transcripts for comparisons. Consistent with the predictions of less effective purifying selection in asexual species, we found that asexuals accumulated nonsynonymous mutations at a higher rate than sexuals (permutation ANOVA: gene effect, species pair effect, reproductive mode effect, and interaction between pair and mode all *P* < 0.001; fig. 6). We additionally ran a simpler three-ratio model that estimates one dN/dS ratio for internal branches and one for terminal branches of each reproductive mode, which corroborated these findings (see Supplementary Material online).

### Increased Substitution Rates at Synonymous Sites in Asexuals

Synonymous substitutions (dS) are commonly assumed to be neutral, and are used to scale nonsynonymous substitutions in the above-mentioned analyses. However, purifying selection can also act on synonymous sites, because different synonymous codons influence for example the speed and accuracy of translation (Hershberg and Petrov 2008). As a consequence, the frequencies by which different codons are used can differ (codon usage bias). We compared the effective number of codons (Enc), a quantitative estimate for the level of codon usage bias, as well as synonymous substitution rates (dS) between sexual and asexual *Timema* pairs. *Timema* featured Enc values that indicate weaker codon usage bias than in animals with large population sizes (e.g., nematodes), but the bias level was similar for species with different reproductive modes (gene effect *P* < 0.001, species pair effect *P* < 0.001, reproductive mode *P* = 0.241, and interaction between pair and mode all *P* = 0.928; permutation ANOVA; supplementary fig. 3, Supplementary Material online). Estimates of dS were systematically elevated in asexuals (gene effect, species pair effect, reproductive mode, and interaction between pair and mode all *P* < 0.001; permutation ANOVA; supplementary fig. 6, Supplementary Material online).

**Fig. 7.**
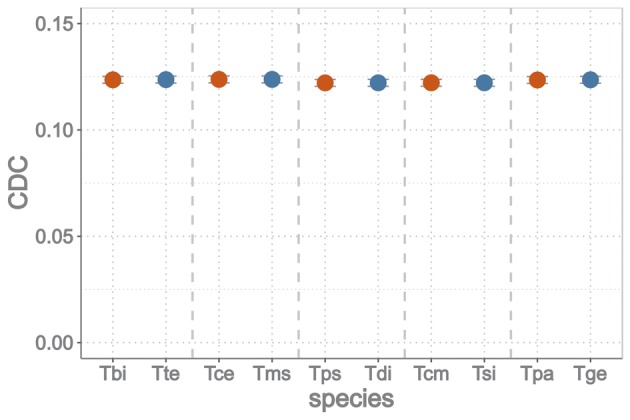
Means (and 95% CI) of the codon deviation coefficient (CDC) of sexual (in red) and asexual (in blue) *Timema* species. Tbi: *T. bartmani*, Tte: *T. tahoe*, Tce: *T. cristinae*, Tms: *T. monikensis*, Tps: *T. poppensis*, Tdi: *T. douglasi*, Tcm: *T. californicum*, Tsi: *T. shepardi*, Tpa: *T. podura*, Tge: *T. genevievae*. Species pairs are ranked by age of the asexual lineage (youngest to oldest).

We tested if the difference in dS between sexuals and asexuals stems from different effectiveness of selection on codon usage bias using the Codon Deviation Coefficient (CDC) as a metric (Zhang et al. 2012). This metric calculates the deviation from expected codon usage bias and accounts for per-gene background nucleotide composition, thus allowing for cross-species comparisons. A lower CDC value would indicate less effective selection on codon usage bias (see Materials and Methods), but we did not detect any differences in CDC value between reproductive modes (gene effect *P* < 0.001, species pair effect *P* < 0.001, reproductive mode effect *P *= 0.595, interaction between pair and mode *P *=* *0.991; permutation ANOVA; fig. 7). Thus, elevated substitution rates at synonymous sites in asexuals are unlikely to stem from less effective selection on codon usage bias in asexuals than sexuals.

### Evaluation of the Consequences of Asexuality Over Time

Given the age variation of the asexual *Timema* species (fig. 2), possible consequences of asexuality can be studied over a range of recent to old asexual organisms. For all of the measured effects of asexuality, lineage age should a priori only impact the consequences of arrested gBGC, because the return to equilibrium base composition should happen gradually over time. Indeed, the effect of arrested gBGC on GC composition was stronger for older than younger asexual *Timema* species (Pearson's product-moment correlation; *t* = 3.29, cor 0.88, *P* = 0.046, supplementary fig. 4, Supplementary Material online). There was no age effect for dN/dS (Pearson's product-moment correlation; *t* = -1.03, cor -0.51, *P* = 0.686), pN/pS (Pearson's product-moment correlation; *t* = -1.23, cor -0.13, *P* = 0.829) and dN estimates (supplementary fig. 5, Supplementary Material online), but surprisingly, dS increased with age of asexuality (Pearson's product-moment correlation; *t* = 5.83, cor 0.96, *P* = 0.010, supplementary fig. 6, Supplementary Material online). However, any correlation (or absence thereof) should be interpreted carefully, as there are only five data points and correlates might be driven by only one old “outlier” asexual (*T. genevieve*).

## Discussion

The absence of recombination is predicted to be associated with reduced GC content (because gBGC does not occur), reduced effectiveness of selection on short and long evolutionary timescales, and affect levels of neutral genetic variation segregating in populations (Muller 1964; Hill and Robertson 1966; Felsenstein 1974; Marais 2003; Keightley and Otto 2006). Using multiple, independently evolved asexual lineages, we provide for the first time empirical evidence for all of these predicted consequences, in full agreement with theory.

gBGC influences the base composition in sexual *Timema* stick insects, but is unlikely to occur (or at least is strongly reduced) in asexuals. Consistent with theory, the effect of arrested gBGC on base composition is stronger in older asexual *Timema* species compared with their sister species (supplementary fig. 4, Supplementary Material online). These findings represent the first documented case of arrested gBGC in asexual eukaryotes. Although there is extensive variation in GC content and in the strength of gBGC among sexual *Timema* species, we consistently detect lower levels of gBGC in the asexual sister lineages. Given this lineage-specific variation, sister-pair comparisons are essential to detect arrested gBGC in asexual species. This might be why all evidence for arrested gBGC in the absence of recombination thus far stems from nonrecombining parts of genomes and selfers, where appropriate comparisons are available (Glémin et al. 2006; Spencer et al. 2006; Duret and Galtier 2009; Glémin and Galtier 2012).

Asexual stick insects exhibit lower levels of segregating genetic variation within populations as compared with sexuals. This finding suggests that asexual *Timema* species are more homozygous than their sexual counterparts, as intra-individual heterozygosity should otherwise generate high genetic variation at individual loci, at least in the old asexual species such as *T. genevievae*. Low genetic diversity in asexuals is also expected because of background selection. Although asexual *Timema* populations are far from being genetically uniform, there are 8-fold more transcripts containing polymorphisms in sexual populations and within these transcripts, variants are twice as frequent (fig. 4). Thus far, comparative studies investigating segregating polymorphisms in sexual and asexual populations are complicated by confounding effects like hybridization events and/or found no differences of the amount of genetic variation between reproductive modes, likely because of young asexual lineage ages (Hollister et al. 2015; Ament-Velásquez et al. 2016). Our findings of reduced levels of standing genetic variation in asexual compared with sexual *Timema* sister species is direct evidence that reproductive mode can strongly influence genetic diversity in natural populations.

Purifying selection is less effective in asexual than sexual *Timema*, resulting in more deleterious variants segregating in populations and a higher rate of deleterious mutation accumulation in the long term. The ratio of nonsynonymous to synonymous segregating variants is approximately two times higher in asexual than sexual populations (range 1.1–2.4, depending on the species pair). In spite of these differences, there is currently no evidence that the average fitness of asexual *Timema* females is lower than the fitness of sexual females, but fitness differences may require more time to build up and become measurable. Indeed some of the deleterious variants become fixed over time as a consequence of drift, resulting in a faster rate of deleterious mutation accumulation in asexual than sexual *Timema* species, with *ω* (dN/dS) on average being 1.2 times higher in asexuals than sexuals (fig. 6).

It is important to note however that *ω* ratios are highly variable among genes (variance = 0.043, range 0.001–0.998), with sometimes opposite effects (*ω*_sex_ *>* *ω*_asex_) to the general pattern. Thus, given this variance, inferring overall genomic patterns from studies that are based on small gene numbers might result in arbitrary and wrong conclusions. Indeed most previous studies on mutation accumulation in asexual metazoans, including a previous study in *Timema*, are based on few genes (Johnson and Howard 2007; Neiman et al. 2010; Henry et al. 2012; Ollivier et al. 2012; Hartfield 2016). To date, only three studies exist that use genome-scale analyses to address mutation accumulation in asexuals, with mixed results (Hollister et al. 2015; Ament-Velásquez et al. 2016; Brandt et al. 2017). Investigated asexual lineages were either very young, and no difference in the effectiveness of purifying selection between reproductive modes was found (Ament-Velásquez et al. 2016). Or very old (tens of millions of years) and accumulating deleterious mutations at lower rates than sexual species, opposite to theoretical predictions (Brandt et al. 2017). In addition to the present study, there is thus far only one study in selfing (but functionally asexual) plants that converged with theoretical predictions of more effective purifying selection in sexual taxa compared with asexuals (Hollister et al. 2015).

Our results also suggest that the reduced effectiveness of selection in asexuals may result in an increase of transcriptional errors (or transcriptional noise). Indeed, an interesting and unexpected finding is that asexual species harbor more variants of orthologous transcripts than sexuals. Analyses using genomic data suggest that these variants do not stem from duplicated genes in the genomes. Moreover, the majority of variants is unlikely to represent different haplotypes, given that they vary considerably in the length of the ORF and the extremely low levels of segregating polymorphism in populations of *Timema* asexuals. Additional studies are required to corroborate our interpretation of higher transcriptional error rates, reduced stability or more frequent posttranscriptional alteration of transcripts in asexuals.

There is evidence that selection can also act on synonymous sites (Lawrie et al. 2013), for example through altering the frequencies by which different codons are used (Duret 2002; Hershberg and Petrov 2008). Interestingly, rates of synonymous substitutions are increased in asexual *Timema*, compared with sexuals. The difference between sexuals and asexuals further increases with the age of asexual lineages. This pattern could be indicative of less effective selection on codon usage bias in asexual than sexual *Timema*. However, while there is weak codon usage bias overall, we could not detect differences in the strength of the bias between sexual and asexual species. This is possibly because evolution of codon bias might be too slow such that differences are too subtle to be detectable in the relatively “young” *Timema* species. Another mechanism that could potentially influence rates of synonymous substitutions is gBGC. gBGC arrest in asexuals results in a sudden change in equilibrium GC3, which under certain conditions is expected to increase substitution rates (Bolívar et al. 2016). Moreover, increased synonymous substitution rates in asexuals could also be caused by increased mutation rates.

In conclusion, this is the first genome scale study on asexual evolution that documents consequences for gBGC, polymorphism levels and effectiveness of purifying selection. This study is particularly robust, because the *Timema* system allows us to utilize evolutionary replicates of independent sexual–asexual sister pairs with different asexual lineage ages. It remains unknown and a challenge for future studies to determine if the accumulation of deleterious mutations may eventually result in the extinction of asexual *Timema* lineages and thereby contribute to the long-term maintenance of sex in this system.

## Materials and Methods

### Taxon Sampling and Sequencing

*Timema* individuals from ten different species (five sexual and five asexual) were collected in California, USA in spring 2014. For details on the sampling locations see supplementary table 3, Supplementary Material online. Prior to RNA extraction, animals were fed with artificial medium for two days to avoid contamination with gut content and then frozen at -80 °C. Total RNA was extracted from whole bodies of a pool of three individuals from the same species and location. This was done by first freezing the individuals in liquid nitrogen, followed by addition of Trizol (Life Technologies) and mechanical bead crushing (Sigmund Lindner). The homogenized tissue was then treated with Chloroform and Ethanol and the aqueous layer transferred to RNeasy MinElute Columns (Qiagen). Following the RNeasy Mini Kit protocol, potential DNA in the sample was digested, RNA eluted into water and stored at -80 °C. RNA quantity and quality was measured using NanoDrop (Thermo Scientific) and Bioanalyzer (Agilent). RNA extracts were pooled and fragmented to 120 nt for strand-specific library preparation. Single-end sequencing with a read length of 100 bp was performed on a HiSeq2000 platform at the CIG (Centre of Integrative Genomics, Lausanne, Switzerland). CutAdapt was used to remove adapter sequences from the raw reads (Martin 2011). Reads longer than 80 bp were then quality trimmed using trimmomatic v 0.36 (Bolger et al. 2014) (first clipping leading or trailing bases with a phred score of <10 from the read, before using a sliding window from the 5′ end to clip the read if four consecutive bases had an average phred score of <20). Following quality trimming any reads <80 bp in length were discarded. For assembly, reads found to contain adapter sequence were not used, but for mapping all trimmed reads were used.

### Assembly and Assembly Cleaning

All available trimmed reads per species were pooled and used as input for assembly with Trinity with the addition of the following option: –min_kmer_cov 2 (v 2.2.0; Grabherr et al. 2011). Assembly often results in an unrealistically high number of transcripts. To remove likely erroneous contigs, we applied a minimum expression filter following Moghadam et al. (2013) and Harrison et al. (2015). We mapped the pooled reads for each sample separately back to the assembly with RSEM v1.2.20 using bowtie2 v2.2.4 (Li and Dewey 2011; Langmead and Salzberg 2012). Ambiguously mapping reads were assigned to the most likely transcript by RSEM. We discarded contigs shorter than 300 bp and with low coverage (reads per kilobase per million mapped, RPKM ≤ 2). After filtering, all assemblies had a comparable number of isotigs and N_50_ (supplementary table 1, Supplementary Material online). For subsequent analyses in this study, the longest isoform per gene/graph was selected. ORFs were identified from the filtered transcriptome set using transdecoder v2.1 running TransDecoder.LongOrfs and TransDecoder.Predict (with strand-specific option). In cases where multiple ORFs were predicted for a single transcript the longest ORF per transcript was selected (supplementary table 1, Supplementary Material online).

### Annotation and Contamination Filtering

NCBI’s BLAST client (v. 2.2.30+) was used to BLAST local versions of the nt (using blastN, default options except -task blastn, -max_target_seqs 10) and nr (using blastX, default options except, -max_target_seqs 10) databases (downloaded: 07/08/2016). BLAST hits were first filtered so that any hit with an *e*-value >0.0000001 was discarded. Contigs were assigned to a Domain if ≥50% of BLAST hits came from a domain. In the event of a tie, the taxa with the highest *e*-value was used as a tiebreaker. Contigs annotated as “noneukaryote” were discarded prior to submission to GenBank (the number of discarded transcripts ranged from 64 to 133 among transcriptomes).

### Orthologs

To identify orthologous sequences shared by any combination of the ten species, OMA v1.0.6 (Altenhoff et al. 2013) was run on the identified ORF protein sequences with default parameters and fixed species tree (fig. 1). Further, orthologs were selected that were shared between all *Timema* species, yielding 3,010 orthologous groups (with one sequence per *Timema* species in each group). To identify the variants of these orthologs from each of the ten transcriptome assemblies, a custom script, provided by Clément Train, a member of the OMA development team, was used (see Code availability).

### Alignments

Alignments of the orthologs shared between sister species were generated to calculate divergence estimates. Alignments were also generated for each of the 3,010 ten-species orthologs for analyses of dN/dS and CDC. For all types of alignments, orthologous sequences were aligned on the protein level using M-coffee for a consensus alignment from multiple software (clustalw2_msa muscle_msa kalign_msa mafftgins_msa t_coffee_msa) implemented in t-coffee v11.00.8 (Wallace et al. 2006). In almost all cases, all methods converged to a similar alignment, as indicated by a quality score > 75. Resulting protein alignments were back-translated into nucleotide alignments using t-coffee and curated with Gblocks v0.91b (type = codons; minimum block length set to 4; Talavera and Castresana 2007) to remove gap-rich and poorly aligned regions.

### Distance Estimates

Pairwise Jukes–Cantor distances of curated alignments from orthologous sequences between sister species were calculated using the distmat program from the EMBOSS suite v6.6.0.0 (Rice et al. 2000).

### gBGC

Ancestral GC3 was estimated for all nodes of the *Timema* tree for the 3,010 orthologs using the NHML program (Galtier and Gouy 1998). This method makes use of a nonhomogeneous and nonstationary Markov model of DNA evolution to estimate branch-specific equilibrium GC-content in a maximum-likelihood framework (Romiguier et al. 2010; Figuet et al. 2014). The substitution mapping procedure was used to calculate the number of synonymous substitutions in the terminal branch of each *Timema* species, and categorized them into weak-to-strong (A/T → G/C), strong-to-weak (G/C → A/T) and GC-conservative (others; Dutheil et al. 2012; Romiguier et al. 2012). This consisted in first fitting the YN98 codon model, as implemented in the bppML program (Dutheil and Boussau 2008), to each alignment of protein-coding sequences. The estimated model parameters were then used to map substitutions on each branch of the tree in the empirical Bayesian framework. The total number of substitutions in a branch for a given category was obtained by summing substitution counts across genes.

### Polymorphism (SNPs)

To identify polymorphism from SNP data, we mapped reads from a pool of three females from the same species and population back to the 3,010 ten-species orthologs (that contained only one “best” variant for each ortholog per orthologous group) with RSEM using bowtie2 with default parameters and –fragment-length-mean 200 –fragment-length-sd 100 (Li and Dewey 2011; Langmead and Salzberg 2012). Samtools v1.2 was used to sort the mapped reads and samtools mpileup to identify SNPs. Resulting polymorphic sites were filtered using VarScan v2.3.2 mpileup2snp with a 20-fold minimum coverage cutoff, 10% minor allele freq and a minimum average phred quality of 20 (following Hollister et al. 2015). To check for mapping bias generated by the presence of variants for certain orthologs, mapping quality and read count was extracted from the RSEM output table and bam files for both the ten-species ortholog set that included the best variant only and a set that included all variants.

### Divergence *ω* (dN/dS) Analyses

Curated back-translated nucleotide alignments of the 3,010 ten-species orthologs were used as input for dN/dS ratio estimates. To estimate gene-specific *ω* (dN/dS) ratios, we first calculated per-gene branch-lengths using RAxML v8.2.8 (Stamatakis 2014) given a fixed, unrooted species tree and the GTRCATI model. Gene-specific branch-lengths, respective alignments and the fixed, unrooted species tree were then parsed as input for codeml, which is included in the paml v4.8 package (Yang 2007), using a custom script that also subsequently collected the output for each gene (see Code availability). We ran both a “free” model, allowing one *ω* value for every branch, and a “three-rate” model, allowing one rate for all asexual and one for all sexual terminal branches as well as one rate for internal (sexual) branches to separate mutations occurring at terminal branches from internal ones. Gene and branch-specific *ω* values were further analyzed with R (Team and Others 2013). We restricted analyses to transcripts with overall *ω* values *ω* < 1 and dS ≠ 0, to capture only transcripts that are under purifying selection. Within this gene set, we removed 200 orthologs that had no *ω* variation between species, leaving 2,487 orthologs to compare. Here, we did not analyze positive selection on a *ω* > 1 gene set, because this would require an entirely different approach that would be based on branch-site-specific models. Given that in the current study, we only used orthologs shared across the ten species, it seems unlikely that we could detect consistently adaptive signals.

### Codon Usage Bias

To infer codon usage bias we calculated the effective number of codons (Enc) per gene using codonW v1.4 (Wright 1990; Peden 2005). Enc quantifies how strongly codon usage deviates from equal usage of all synonymous codons, taking values from 20 (each amino acid is encoded by only one codon exclusively) to 61 (equal use of all possible alternative codons). Following, Enc values below 61 indicate codon usage bias.

We estimated signatures of relaxed selection on synonymous sites using the CDC (Zhang et al. 2012). CDC estimates the expected codon usage from observed GC and purine contents and calculates the deviation from observed codon usage. CDC ranges from 0 (no detectable [“relaxed”] selection on codon usage) to 1 (effective selection on codon usage). We calculated CDC using the same alignments as used for *ω* analyses.

### Polymorphism (pN/pS) Analyses

We used the SNP data (details above under “polymorphism”) to identify nonsynonymous and synonymous segregating polymorphism in the ten *Timema* populations. For this, we identified the fold-degenerate position of resulting filtered nucleotide variants using a custom script following Li et al. (1985) from which pN, pS and (pN/pS) was calculated per gene (see Code availability).

### Statistics

For statistical tests on dN/dS, dS, Enc and CDC estimates we used a permutation ANOVA (Manly 1997) with 5,000 bootstrap replicates (script available in Henry et al. 2012). For other analyses, statistical methods are given in the text.

### Data Availability

Raw reads are deposited in SRA under accession codes SRR5748941–SRR5749000. The transcriptome assemblies are deposited at DDBJ/EMBL/GenBank under the BioProject PRJNA380865 with the following accession codes: GFPP00000000, GFPR00000000, GFPS00000000, GFPT00000000, GFPU00000000, GFPV00000000, GFPW00000000, GFPX00000000, GFPY00000000, and GFPZ00000000. Data will be publically released upon acceptance.

### Code Availability

The custom scripts used in this study are deposited at https://github.com/jensbast/TimemaConsOfAsex/, last accessed March 16, 2018.

## Supplementary Material

Supplementary data are available at *Molecular Biology and Evolution* online.

## Supplementary Material

Supplementary Data
