# Supplementary material for: Consequences of Asexuality in Natural Populations: Insights from Stick Insects
Source: Mol Biol Evol. 2018 Apr 5;35(7):1668–77. doi: 10.1093/molbev/msy058 (PMC5995167; doi:10.1093/molbev/msy058)
Supplement: Supplementary Data [file msy058_supp.zip › supplementary.pdf]

Supplementary

Three-rate model

We used a three-ratio model, which calculates one dN/dS ratio for internal branches and one for terminal branches leading to asexual species and one for terminal branches leading to sexual species. In this way, only mutations occurring after the split of sexuals and asexuals are taken into account. After filtering data for which ω did not converge (0.0001 or 999 ω values for both terminal and internal branches) or where ω<1, 1647 genes remained for this model. We detected a small but significant difference for ω, with a faster rate of mutation accumulation for asexual *Timema* species (paired Wilcox test $P < 0.01$; median $\Delta_{sex\text{-}asex}$ = -0.008). This indicates that asexual *Timema* species overall accumulate non-synonymous mutations at a higher rate.

Variant coverage estimation

Genomic reads from SRA (SRR5248877 - SRR5248936) were mapped to the transcriptome assemblies using Bowtie2 (v2.2.4). We used the local alignment mode to allow reads to be soft-clipped when mapping, since genomic reads contain intronic sequence not found in the transcriptomes. Coverage for each ortholog and ortholog variant was obtained using Bedtools (v2.26.). Coverage for each ortholog and its variants were summed together.

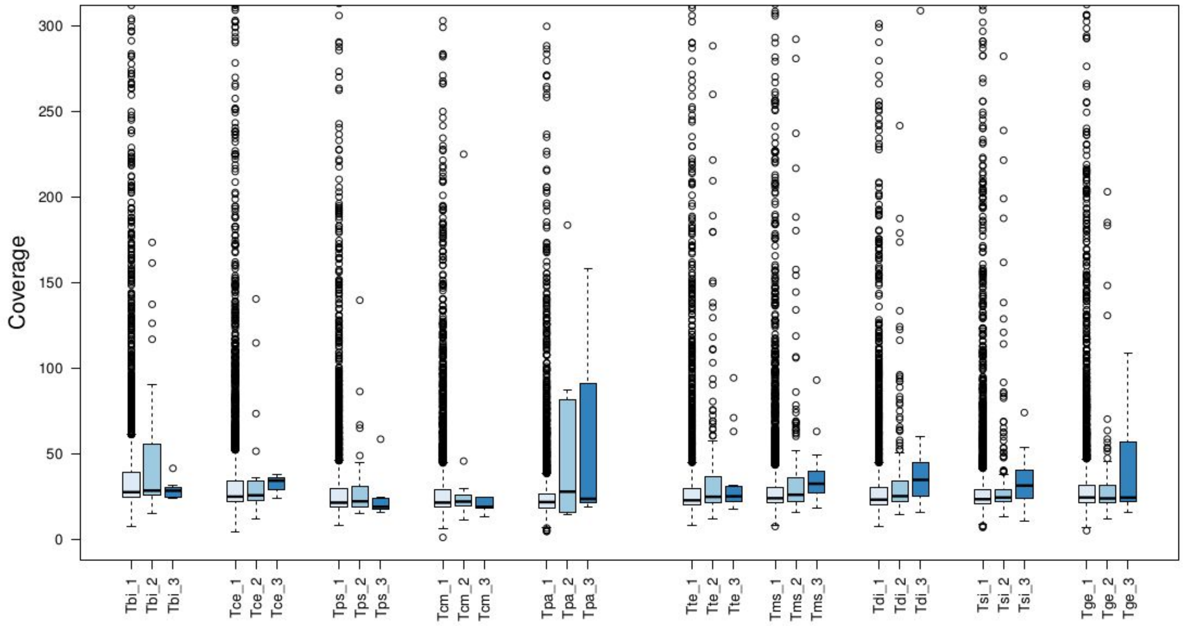

Supplementary Figure 1: Orthologous variants do not stem from gene duplicates. Genomic per-base read coverage for orthologs with one, two or three variants per species. Only orthologs or ortholog variants with coverage between 1-2000x were used for plotting (discarding a total of 83 ortholog or ortholog variants). Orthologs with ≥ 4 variants were a maximum of 14 per species and were also not plotted. Tbi: *Timema bartmani*, Tte: *T. tahoe*, Tce: *T. cristinae*, Tms: *T. monikensis*, Tps: *T. poppensis*, Tdi: *T. douglasi*, Tcm: *T. californicum*, Tsi: *T. shepardi*, Tpa: *T. podura*, Tge: *T. genevievae*.

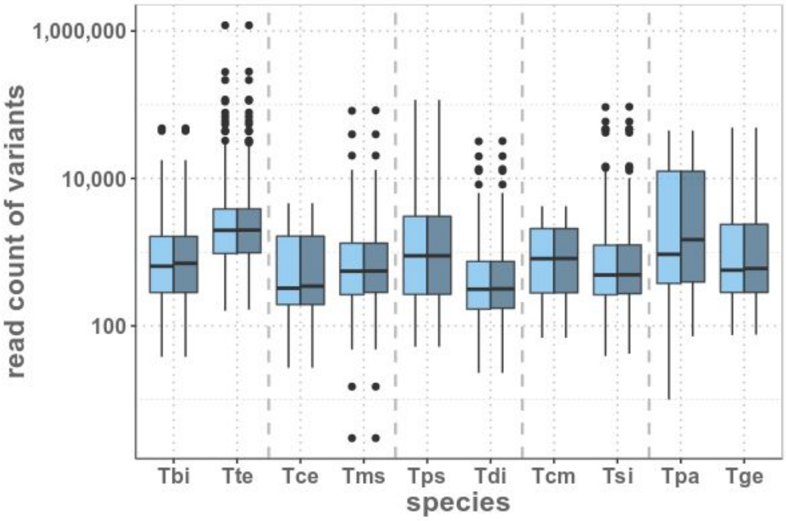

Supplementary Figure 2: Reads corresponding to variants of specific orthologs mapped to the best ortholog if the variants were excluded. Read counts for the best ortholog only (in light blue) and summed counts for reads mapping to all variants, including the best ortholog (in dark blue) were similar (paired t-test, t = -0.023, df = 2704, *P* = 0.982). As expected given the larger number of variants in asexuals than sexuals, mapping quality was somewhat lower in asexuals than sexuals, however not significantly so (asexuals: range 99.84 - 99.89, sexuals: range 99.86 - 99.91; paired t-test, t = 1.3, df = 7.9, *P* = 0.215). Tbi: *Timema bartmani*, Tte: *T. tahoe*, Tce: *T. cristinae*, Tms: *T. monikensis*, Tps: *T. poppensis*, Tdi: *T. douglasi*, Tcm: *T. californicum*, Tsi: *T. shepardi*, Tpa: *T. podura*, Tge: *T. genevievae*.

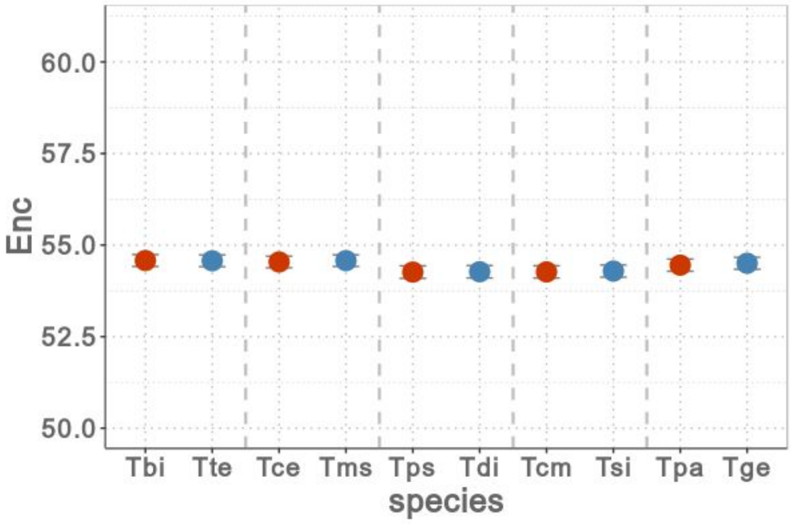

Supplementary Figure 3: *Timema* species exhibit codon usage bias and the effective number of codons used do not differ between sexuals asexuals (means and 95% CI). Usage of fewer than 61 codons indicates that some codons are used more frequently than others. Note that the axis does not start at zero. Tbi: *Timema bartmani*, Tte: *T. tahoe*, Tce: *T. cristinae*, Tms: *T. monikensis*, Tps: *T. poppensis*, Tdi: *T. douglasi*, Tcm: *T. californicum*, Tsi: *T. shepardi*, Tpa: *T. podura*, Tge: *T. genevievae*.

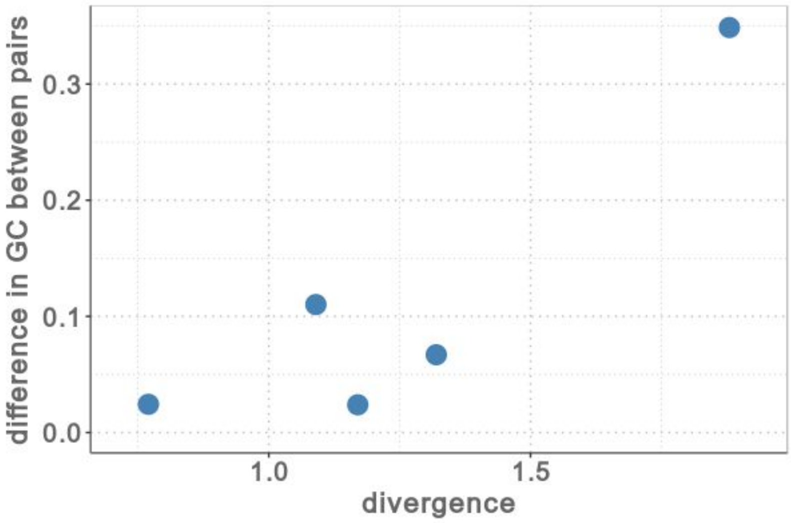

Supplementary Figure 4: Base composition difference increases with asexual lineage age. Euclidian distance between sister-species for GC3% means and GC3 std correlated with divergence between the sexual and asexual species within a pair.

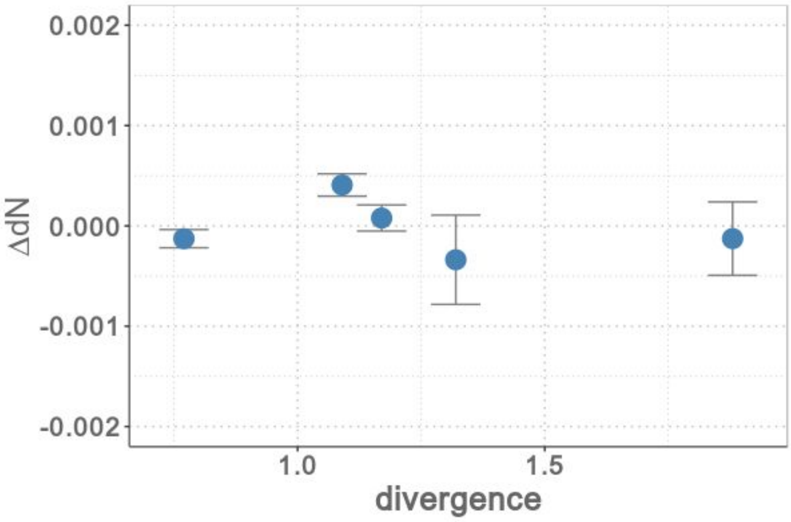

Supplementary Figure 5: The rate of dN does not increase with asexual lineage age (Pearson's product-moment correlation; t = -0.450, cor -0.249, *P* = 0.686). Means (and 95% CI) of $\Delta dN_{asex\text{-}sex}$ correlated with divergence between the sexual and asexual species within a pair.

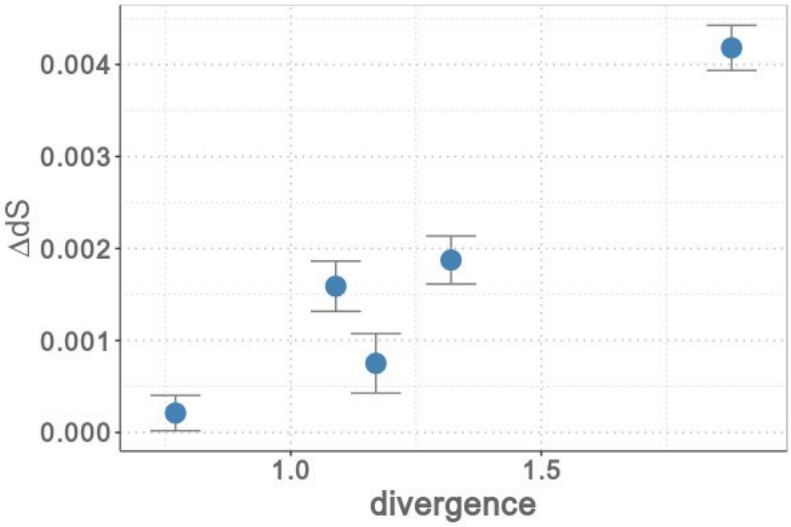

Supplementary Figure 6: The rate of dS increases with asexual lineage age. Means (and 95% CI) of $\Delta dS_{asex\text{-}sex}$ correlated with divergence between the sexual and asexual species within a pair.

Supplementary Table 1: Statistics for the quality filtered assemblies.

species	pair	repr mode	No of transcripts	Longest transcript length (bp)	N50	No of ORFs
T. bartmani	**1**	sex	31747	21998	1835	7741
T. cristinae	**2**	sex	34177	29940	1729	7703
T. californicum	**4**	sex	32416	28616	1737	8026
T. douglasi	**3**	asex	37652	24333	1530	8910
T. genevievae	**5**	asex	44740	28013	1499	9409
T. monikensis	**2**	asex	43326	27229	1512	10061
T. podura	**5**	sex	45655	30228	1301	9392
T. poppensis	**3**	sex	32167	30132	1756	7136
T. shepardi	**4**	asex	41642	29999	1493	10196
T. tahoe	**1**	asex	42372	24302	1640	9634
**mean sexual species**			35232	28183	1672	8000
**mean asexual species**			41946	26775	1535	9642

Supplementary Table 2: Number of orthologs per species pair.

Species pair	Number of pairwise orthologs
T. bartmani - T. tahoe	5908
T. cristinae - T. monikensis	5806
T. californicum - T. shepardi	5754
T. poppensis - T. douglasi	5476
T. podura - T. genevievae	5329

Supplementary Table 3: Coordinates of the sampling locations.

Species		latitudes	longitudes
T. bartmani		34.170000	-117.002017
T. californicum		37.222860	-122.087231
T. cristinae		34.522300	-119.831283
T. douglasi		39.200783	-123.293933
T. genevievae		38.995783	-122.925767
T. monikensis		34.102933	-118.861783
T. podura		33.797603	-116.776878
T. poppensis		36.996433	-121.717783
T. shepardi		38.982550	-123.469750
T. tahoe		38.761011	-120.160053
